# Genetic Diversity and Population Structure of European Soybean Germplasm Revealed by Single Nucleotide Polymorphism

**DOI:** 10.3390/plants12091837

**Published:** 2023-04-29

**Authors:** Zoe Andrijanić, Nelson Nazzicari, Hrvoje Šarčević, Aleksandra Sudarić, Paolo Annicchiarico, Ivan Pejić

**Affiliations:** 1 Centre of Excellence for Biodiversity and Molecular Plant Breeding, Svetošimunska Cesta 25, 10000 Zagreb, Croatia; zandrijanic@agr.hr (Z.A.); aleksandra.sudaric@poljinos.hr (A.S.); ipejic@agr.hr (I.P.); 2 Research Centre for Animal Production and Aquaculture, Council for Agricultural Research and Economics (CREA), Viale Piacenza 29, 26900 Lodi, Italy; paolo.annicchiarico@crea.gov.it; 3 Faculty of Agriculture, University of Zagreb, Svetošimunska Cesta 25, 10000 Zagreb, Croatia; 4 Agricultural Institute Osijek, Južno Predgrađe 17, 31000 Osijek, Croatia

**Keywords:** soybean, genetic diversity, genetic resources, population structure, single-nucleotide polymorphism

## Abstract

Soybean is the most grown high-protein crop in the world. Despite the rapid increase of acreage and production volume, European soybean production accounts for only 34% of its consumption in Europe. This study aims to support the optimal exploitation of genetic resources by European breeding programs by investigating the genetic diversity and the genetic structure of 207 European cultivars or American introductions registered in Europe, which were genotyped by the SoySNP50K array. The expected heterozygosity (H_e_) was 0.34 for the entire collection and ranged among countries from 0.24 for Swiss cultivars to 0.32 for American cultivars (partly reflecting differences in sample size between countries). Cluster analysis grouped all genotypes into two main clusters with eight subgroups that corresponded to the country of origin of cultivars and their maturity group. Pairwise F_st_ values between countries of origin showed the highest differentiation of Swiss cultivars from the rest of the European gene pool, while the lowest mean differentiation was found between American introductions and all other European countries. On the other hand, F_st_ values between maturity groups were much lower compared to those observed between countries. In analysis of molecular variance, the total genetic variation was partitioned either by country of origin or by maturity group, explaining 9.1% and 3.5% of the total genetic variance, respectively. On the whole, our results suggest that the European soybean gene pool still has sufficient diversity due to the different historical breeding practices in western and eastern countries and the relatively short period of breeding in Europe.

## 1. Introduction

Soybean is the most grown high-protein crop in the world, with beans containing high levels of protein (39–42%) and oil (19–22%) [1,2]. It is grown on about 121 million hectares worldwide, and the global annual production is estimated at 334 million tons (FAOSTAT, 2021). The largest producer is Brazil, followed by the United States and Argentina, which together account for 73% of global soybean production. Asian countries produce about 20% of the world’s soybeans, while Europe accounts for only 3% of the world’s soybean production [3].

Soybean acreage in Europe has nearly doubled over the past decade, and soybeans are now grown on 5.3 million hectares, representing 4.4% of the total global soybean acreage [3]. Most of it is situated in Southeast and East Europe. Despite this rapid increase in acreage and production volume, European soybean production accounts for only 34% of the total 34.4 million tons consumed in Europe [4]. To satisfy the growing interest of European farmers and reduce dependence on imports, it is necessary to continuously improve cultivars in terms of yield, quality, and tolerance to stress factors, but also to increase their adaptability to different environments. There is significant potential for expanding soybean cultivation to new areas in Northern and Central Europe [5,6], as well as in Southeast Europe, where favorable growing conditions already exist.

A basic requirement for successful crop breeding is the continuous provision and exploitation of genetic diversity, which serves as a reservoir of tolerance/resistance traits for crop adaptation to new environmental conditions and the changing climate. The common use of just few elite cultivars by farmers reduces the ability to maintain sufficient crop production under increasingly frequent extreme climatic events and associated environmental stresses. The genetic diversity of many crops declines over time as commercial plant breeding focuses on improving one or a few traits and/or uses only a small number of elite genotypes to develop breeding populations [7]. The study of diversity patterns and the genetic structure of germplasm resources based on molecular characterization data can guide the selection by breeding programs of divergent parents for crosses and the optimal exploitation of genetic resources, especially in the absence of reliable pedigree data [8]. This is the case for soybean in Europe, for which there is little reliable information on the origin or pedigree of soybeans introduced from the 17th century onwards. Early germplasm introductions were from China and Japan, and are still used to some extent nowadays, mainly for developing early maturing cultivars [9]. Although the genetic background of early-maturing soybean is thought to be mainly from North America, there are some Central European breeding programs that have been in place for more than 30 years and incorporate Asian germplasm, such as that in Switzerland [10]. On the other hand, most Southeast European breeding lines and registered soybean cultivars are closely linked to breeding programs from the USA and Canada [11,12].

The development of DNA marker technology, such as simple sequence repeats (SSRs) and single nucleotide polymorphisms (SNPs), has created the technical tools required to evaluate the genetic relationships within soybean germplasm. Due to the minor role of soybean in European agriculture, few studies investigated the genetic diversity of European germplasm [11,13,14,15,16,17]. Using SSR markers, Ristova et al. [13] analyzed genetic diversity and relationships among soybean genotypes representing multiple sources from Southeast Europe and introductions from Western Europe and Canada. All genotypes were clearly separated according to their origin and known pedigrees. Hahn and Wurchum [14] characterized the European soybean germplasm using genome-wide DaRT markers and found that Central European lines were most closely related to Canadian lines and slightly more distant from Chinese and US lines. It was suggested to use Asian or US lines to further increase the genetic diversity for long-term breeding success. Žulj Mihaljević et al. [11] investigated the diversity and structure of 97 European commercial soybean cultivars using SSR markers. Lower diversity was found compared to Asian and American germplasm, and structure was mainly influenced by the environmental adaptation of the founding germplasm determined by its maturity group. However, the effect of geographic origin on marker-based clustering of European commercial germplasm was weaker, which was explained by the short history of soybean cultivation in Europe and the use of common elite germplasm in most breeding programs. Yao et al. [17] compared the diversity of early maturity Chinese and European elite soybeans using SSR and SNP markers, and found that the level of genetic diversity was similar between the two populations. The European population was markedly structured by maturity group, which was less clear in the Chinese population. In contrast, Saleem et al. [16] showed that the structure of SNP diversity was related to the geographical origin but not to the maturity group in a soybean germplasm collection relevant to breeding in Europe. Previous studies [9,11,14,16], have revealed a relatively narrow genetic base of the European soybean germplasm, which could be explained by the fact that only a small number of ancestors from Canada, the USA, Japan, and China were used for breeding in Europe [13,14,15]. The starting material used by European soybean breeders probably had low genetic diversity itself, as shown by studies on US and Canadian initial parent germplasm [18,19,20]. Reduced genetic diversity also derived from the fact that only maturity groups 000 to II were suitable for cultivation in Europe, as flowering and maturity of soybean depend on photoperiod and temperature [15].

Although some insights into the genetic background of European soybean germplasm have been gained recently, there are still many uncertainties about its origin, diversity, and genetic structure. This work focuses on the molecular diversity of a geographically broad germplasm collection of 207 historical and modern soybean cultivars (MG000 to MGII) relevant to commercial production and breeding under Southeast European conditions. It aims to support the optimal exploitation of genetic resources by European breeding programs by (a) assessing the genetic diversity and the genetic structure of the collection based on a large number of SNP markers, and (b) comparing genetic diversity parameters among country breeding programs and maturity groups.

## 2. Results

### 2.1. Marker Quality and Filltering

The germplasm collection, consisting of 207 soybean cultivars, was genotyped by the Soy50k Illumina Bead Chip [21], resulting in 52,041 SNP recorded markers. After filtering for monomorphic markers, markers with ≥20% missing rate, and those with minor allele frequency < 0.05, we retained 20,541 SNPs for further statistical analysis, with an average heterozygosity of 1.7% across all loci.

### 2.2. Genetic Diversity

The expected heterozygosity (H_e_), polymorphic information content (PIC), and minor allele frequency (MAF) for the entire collection were 0.34, 0.27, and 0.25, respectively (Table 1). The same parameters were used to quantify the diversity within countries of origin and maturity groups. H_e_ ranged from 0.24 for Swiss cultivars to 0.33 for American germplasm (Table 1).

PIC varied from 0.19 to 0.26, and MAF from 0.18 to 0.24, displaying a similar variation pattern across countries as H_e_. In general, broader diversity was found within the American introgressions and the Austrian cultivars, while narrower diversity featured the Swiss and Croatian cultivars (Table 1). There were no obvious differences in genetic diversity parameters among maturity groups (Table 1).

### 2.3. Population Structure of the Soybean Collection

**Figure 1:**
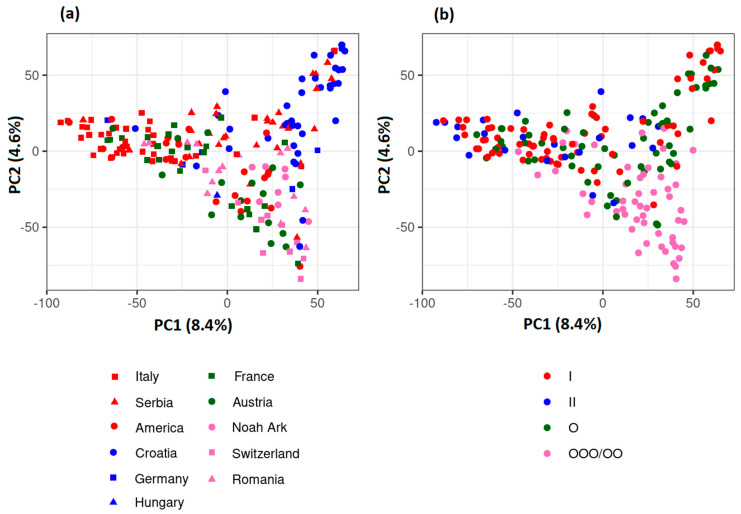
Principal component analysis of 207 cultivars of the European soybean germplasm collection based on (**a**) country of origin and (**b**) maturity groups.

Principal component analysis (PCA) based on the SNP marker data was used to assess the population structure of the collection. The proportion of total genetic variability explained by the first two principal components was 13% (Figure 1). The analysis showed that there was no major population structure with respect to either country of origin or maturity group, although some weak groupings were observed. For example, with few exceptions, the first principal component separated Croatian from Italian and French cultivars, which tended to group together, and the second principal component separated Croatian from Swiss cultivars (Figure 1a). With respect to maturity group, the earliest cultivars (MG000/00) were slightly separated from the cultivars belonging to MG0, MGI and MGII, which tended to group together (Figure 1b).

**Figure 2:**
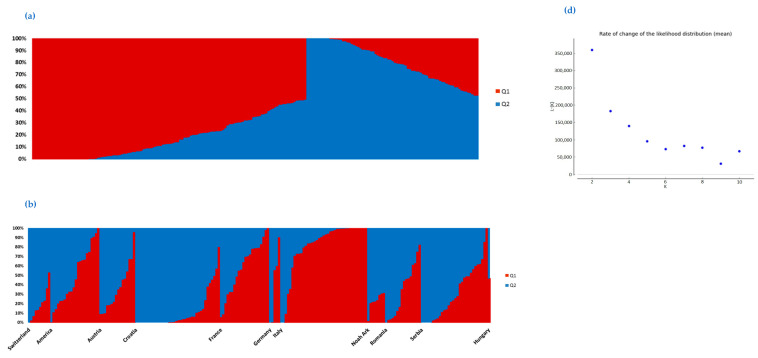

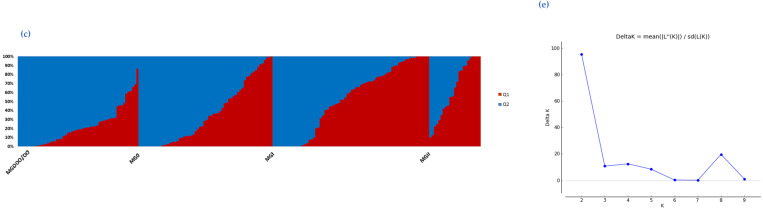
Graphical representation of the proposed structure of the analyzed set of cultivars for k = 2 according to structure analysis. Each cultivar is represented by a vertical column painted in accordance with the Q coefficient and the corresponding source group (**a**). Cultivars are grouped according to the countries of their origin (**b**) and maturity groups (**c**). Rate of change of the likelihood distribution (**d**) and delta K Evanno method (**e**) are shown for each possible K.

Model-based structure analysis revealed two groups (Q1 and Q2), corresponding to the best-fit K-value of 2 (Figure 2). There were 41 cultivars in Q1 and 75 cultivars in Q2, while 91 were considered admixed according to an 80% affiliation rate. Considering the share of ancestral populations by each country (Figure 2, Table S1), the Italian germplasm was the only one that belonged predominantly to ancestral population 1 with 71.8% cultivars in Q1. The French soybean germplasm originated either from ancestral population Q1 or it was of admixed origin. On the other hand, the Croatian and Swiss cultivars belonged predominantly to ancestral population Q2 (with a proportion of 84.2% and 60.0%, respectively, in group Q2). Most Serbian cultivars originated either from ancestral population Q2 (43.3%) or were of admixed origin (50.0%). Cultivars from Noah’s Ark and American introductions were predominantly of admixed origin. In terms of maturity (Figure 2, Table S1), the highest proportion of cultivars from all groups except MG0 were of admixed origin. However, the proportion of cultivars belonging to Q1 gradually increased (and that of cultivars belonging to Q2 decreased) passing from the earliest (MG000/00) to the latest (MGII) group. For comparison, 1.9% and 50.0% of the cultivars belonged to Q1 and Q2, respectively, in the earliest material (MG000/00), while 43.5% of the cultivars belonged to Q1, and only 8.7% to Q2 in the latest group (MGII).

**Figure 3:**
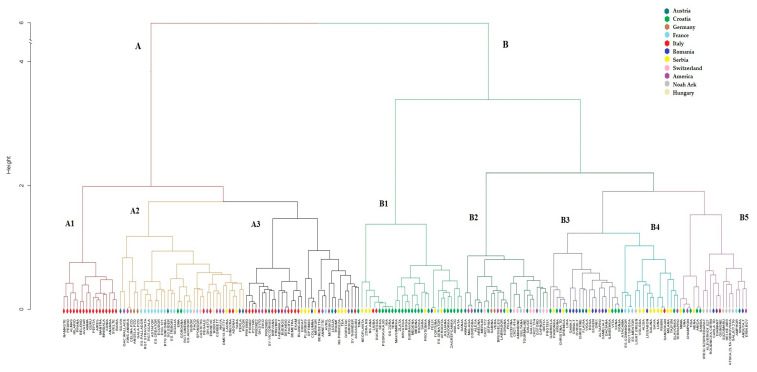
Cluster analysis of 207 soybean cultivars using Ward’s minimum variance method based on Nei’s genetic distances. Colors represent the country of cultivars’ origin.

Cluster analysis revealed two main clusters, consistent with the results from structure analysis (Figure 3, Table S2). Cluster A (*n* = 90) in the dendrogram consisted mainly of Italian cultivars (35/39), French cultivars (15/22), and introductions from America, divided into three subgroups as follows: A1 consisted only of Italian cultivars; A2 mainly of French cultivars and introductions from America; while A3 was dominated by cultivars from Italy, America, and Serbia. The larger cluster B (*n* = 117) consisted mainly of Croatian, Serbian and Romanian cultivars, followed by Austria, America and Switzerland. Subcluster B1, included mainly Croatian and some Serbian cultivars; B2 was dominated by Central European cultivars from Switzerland and Austria, along with some American cultivars; B3 consisted mainly of Southeastern European cultivars from Romania, Croatia and Serbia; B4 contained mainly Serbian cultivars; B5 contained the majority of Noah’s Ark with some Southeastern European cultivars and two introductions from America. With respect to maturity groups (Figure S1, Table S3), cluster A consisted mainly of cultivars with intermediate or late maturity (MGI, MG0 and MGII), with only eight early cultivars (MG000/00), whereas the larger cluster B consisted mainly of cultivars of MG000/00 (46) and MG0 (40), about half as many cultivars of MGI, and only seven cultivars of MGII. Subcluster B2 consisted almost entirely of MG000/00 cultivars.

The dendrogram (Figure 3) shows the existence of nine pairs of highly related (nearly identical) cultivars, as well as of one cluster of four highly related cultivars with mutual genetic distance values below 0.05. The cultivars in seven out of the nine genetically highly similar pairs originated from different breeding programs/countries (Table S4). In most cases, they belong to the same maturity group. In the case of the cluster consisting of four genetically closely related cultivars (Figure 3, cluster B1), all of them originated from the same breeding program.

The analysis of molecular variance (AMOVA) showed that the variation among countries of origin and that among maturity groups explained 9.8% and 4.5% of the total variation, respectively, confirming the fairly modest variation accounted for by these cultivar classification criteria (Table 2).

F_st_, as a measure of population differentiation due to genetic structure, revealed low average differentiation among countries of origin (average F_st_ = 0.128), while differentiation between maturity groups was much lower (average F_st_ = 0.053) (Table 3). The highest average differentiation was observed for Swiss cultivars (F_st_ = 0.22) and the lowest for American cultivars (F_st_ = 0.08), whereas the average F_st_ for other countries ranged from 0.10 to 0.15. In addition to the American cultivars, relatively low average differentiation was also found for Austrian and Serbian cultivars (F_st_ = 0.10). These three groups showed the highest similarity among themselves with the respective F_st_ values ranging from 0.05 to 0.08.

## 3. Discussion

Although soybean has become one of the most important sources of high-protein feed and plant-based food, little is known about the diversity and interrelationships in the European commercial germplasm gene pool. The observed value of 0.34 for gene diversity (expected heterozygosity) of the entire collection is similar to results in Hahn and Würschum [14], who reported a value of 0.33 for Central European soybean germplasm. The value of 0.33 was also found for Chinese germplasm, while a lower value emerged for American germplasm (0.30) [22]. A higher gene diversity value was reported for breeding lines from sub-Saharan Africa (0.41) [23]. Earlier reported PIC values followed a similar pattern for comparison with our results, resulting similar for Chinese and American germplasm [22] and higher for African germplasm [23]. The relatively high gene diversity and PIC values reported in [23] may actually be due to the inclusion of the African panel, some accessions from Asian countries, Brazil, the USA, and Canada, and the fact that only a relatively small number of preselected, highly informative KASP markers were used. Nevertheless, the reported SNP diversity is lower than the microsatellite (SSR)-based diversity reported in previous studies. For example, SSR diversity in Asian [24] and European [11] germplasm was reported to be 0.78 and 0.63, respectively, which is not surprising given the multi-allelic nature of SSR markers [25]. In terms of country of origin, gene diversity was lowest in Swiss cultivars, likely because of high selection pressure for very early ripening and possibly the smaller sample size compared to other countries (Table 1).

We investigated the population structure with different approaches, which provided fairly similar indications. Structure analysis indicated the presence of two subpopulations in the European gene pool, in agreement with previous results for European commercial germplasm based on SSR and SNP markers [11,17]. The PCA ordination revealed the absence of large population structure, but some weak similarity patterns of genotypes according to their country of origin and maturity were observed. In terms of maturity group, the earliest cultivars (MG000/00) were separated from cultivars belonging to MG0 to MGII, which is consistent with Yao et al. [17], who also observed a clear separation of the early maturity group (MG0000 to 00) from the later groups (MG0 to II) within a set of European elite soybean cultivars. The results of the AMOVA showed that the country of origin had stronger influence on population structure than the maturity group, as observed in Saleem et al. [16].

Cluster analysis revealed two main clusters, which corresponded to the two subgroups obtained by structure analysis. These subgroups may be related to the history of soybean introductions to Europe, which took different paths in the historical eastern and western blocks [26]. The smaller group (shown as group A in Figure 3 and Q1 in Figure 2) is dominated by Western countries and consists of three subclusters, where A1 contains only Italian cultivars and A3 consists mainly of Italian, Serbian, and North American cultivars. This is consistent with the study by Saleem et al. [16], who reported a close grouping of Italian and Serbian cultivars with a set of accessions from the United States and Canada. Cluster analysis also revealed the close grouping of French cultivars with North American cultivars, likely due to their American origin or introduction of material from Canada registered in Europe. The clearest separation from the other European cultivars was observed for Italian cultivars, which could be explained by their predominant origin from the breeding program by ERSA (*Agenzia regionale per lo Sviluppo Rurale del Friuli Venezia Giulia*), whose breeding activities started only recently (in 1987) with the main objective to create new cultivars with high grain yield and low level of antinutritional factors, such as the Kunitz trypsin inhibitor [27]. Therefore, this germplasm could be interesting for further breeding in European programs. Cluster analysis also revealed that Croatian cultivars are somewhat isolated from other European cultivars, which is consistent with the separation of these cultivars from Southeast European cultivars in a recent study [11].

In the present study, extremely low genetic distance values (high genetic similarity) based on high number of SNP loci were observed in nine pairs of cultivars and within one cluster consisting of four Croatian cultivars. However, the four Croatian cultivars have been released 3–5 years one after another and had similar but not identical pedigree. All these four cultivars passed the DUS and VCU test and had significant market success in Croatia.

Extremely low genetic distance within soybean germplasm has been observed in a similar study by Fu et al. [28] It is known that breeders sometimes derive several prospective lines out of a good offspring in late generations of inbreeding, and that they can be phenotypically rather distant. Also, use of same or genetically related parents for crosses could lead to low values of genetic distance even among different breeding programs. However, observed low genetic distance between accessions that were bred for and grown in very distant areas might be also the consequence of mislabeled accessions or due to unreliable maintenance in germplasm collections. The germplasm collection used for this study was recently composed through the acquisition of cultivars retrieved from several research and breeding institutions and it might contain a certain number of duplications due to mislabeling. Obtained results will be used to carefully examine and validate suspected genetic relatives or duplicates in the field analysis and through communication with breeders.

Generally, there is very modest information and a lack of tools for public but reliable genotype verification at the international level. The CPVO Variety Finder is a simple, helpful tool (database) to discover some basic information on breeding companies, seed maintainers and providers, as well as synonyms (different commercial names) at European markets, but it is not consistently compiled and maintained. In conclusion, so far there is no reliable referent database of soybean cultivars in Europe that would provide basic morphological, biological, and genetic identification data. Our results might contribute to soybean germplasm management and utilization.

F_st_ estimates showed the high differentiation of Swiss cultivars from the rest of the European gene pool. In addition, Swiss cultivars showed higher differentiation from American germplasm than cultivars from other European countries. The differentiation between Swiss cultivars and Central European germplasm and their weaker relationship with American lines was reported earlier [14] and may partly be due to the early and significant introgression of Asian genetic resources [10], as well as the strong focus on early-maturing genotypes. The lowest mean differentiation was found between the American pool and all other countries, a result that could be explained by the large use of American parent germplasm by European breeders. The adaptive importance of maturity time for national breeding programs was reflected by the grouping of the earliest material MG000/00 in the PCA and the increase of F_st_ as a function of the difference in maturity class. A similar genetic separation of very early material was observed earlier [11], concurrently with signatures of selection for at least two loci involved in photoperiod sensitivity and time to flowering, as a consequence of selection for early flowering when adapting soybean for cultivation in Europe. European breeding programs have only a fraction of the variability available in non-European collections, because only MG000-MGII cultivars can be grown. In soybean breeding, high selection pressure was exerted on maturity loci [15,20]. Low nucleotide diversity in European soybean collections in regions containing known QTLs for seed fatty acids, seed oil, yield components, resistance to biotic stresses, and flowering time [16]. The exploitation of novel early-maturity alleles [29] may partly counterbalance the diversity loss experienced by the European gene pool as a consequence of selection for early maturity. Nonetheless, European germplasm showed genetic diversity compared to American germplasm in this study as a likely consequence of intense exchange of genetic resources among European countries, and between most of them and the United States or Canada [11,30]. Other reasons for the sizeable genetic diversity of the European gene pool are the historical separation between Eastern and Western breeding programs and, to a lesser extent, the relatively short time span of most European breeding program.

Our results can inform the introduction of genetic resources by regional European breeding programs that aim to widen the available genetic diversity for soybean selection. For example, the Swiss germplasm was most divergent within the European gene pool and has particular interest for breeding programs aimed to select very early cultivars. This phenological type is important to expand soybean production to Northern European regions, or for breeding varieties suitable as a second crop in Southern Europe. Our results suggest that genetic diversity of soybean germplasm in Europe is not a limiting factor for further progress in soybean breeding. The introduction of extra-European genetic resources has high interest as well for European breeding programs, especially in the context of climate change, as devised by Haupt and Schmid [31] by means of core collections whose genetic variation emphasizes the tolerance to key abiotic stresses.

## 4. Materials and Methods

### 4.1. Plant Material

The plant material used in this study consisted of 207 soybean cultivars representing the European gene pool with suitable maturity groups ranging from MG000 to MGII (Table 4). Traditional and elite cultivars, collected from breeding companies and reference collections in Croatia (Faculty of Agriculture University of Zagreb, Zagreb, Croatia, Agricultural Institute Osijek, Osijek, Croatia, Croatian Agency for Agriculture and Food, Osijek, Croatia) and Italy (Regional Agriculture Agency of Friuli Venezia Giulia,, ERSA, ERSA, Pozzuolo del Friuli UD, Italy, and Council for Agricultural Research and Economics, CREA, Lodi, Italy) were divided into groups according to the country of origin (origin of breeding program) and maturity group. Additionally, several historical accessions originating from Asia (Russia, China, Japan) obtained from the Noah Ark collection (www.arche-noah.at), and the North and South American continents (Argentina, Canada, and USA) were also included in this investigation because these were introduced in the European soybean breeding program during the past decades. Specific information for plant material is listed in Table S4 and Figure S2.

### 4.2. SNP Genotyping and Data Filtering

Seeds from each accession were sent to TraitGenetics Ltd. in Gaterslaben for genotyping. Single nucleotide polymorphism genotyping was conducted on SoySNP50K Illumina Infinium BeadChips (Illumina, Inc., San Diego, CA, USA) followed by the InfiniumH HD Assay Ultra protocol (Illumina, Inc., San Diego, CA, USA) as described by Song et al. [21]. Genotype data consisted of 52,041 SNPs evaluated on 207 cultivars and further filtered for monomorphic markers, quality (GC > 0.15, GT > 0.5 as per Illumina specification), maximum 20% missing rate for marker, and genotype using *SnpReady* R package. All heterozygous markers were removed, and imputation was performed according to Wright’s method [32].

### 4.3. Statistical Analysis and Genetic Differentiation of Soybean

Cultivars, prior to the analysis itself, were classified into the countries of their origin and maturity groups. Principal component analysis was performed to summarize the genetic structure and variation present in the collection using *popgen* R package [33]. The same package was used for genetic diversity measures (H_e_, PIC, MAF). Genetic diversity measures by country of origin were calculated only for countries represented by 10 or more cultivars. The clustering of cultivars was performed using the R package “stats” and Ward’s minimum variance method based on Nei’s genetic distances. The Bayesian model-based method STRUCTURE, version 2.3.4 [34], was used to imply genetic structure and define the number of groups within the analyzed dataset. For each assumed k, which ranged from 1 to 10, a total of 2 replicates were performed. Each replicate consisted of a burn in a period of 100,000 steps followed by 100,000 Monte Carlo Markov chain iterations assuming the mixed model and correlated allele frequencies. The most likely number of groups (k) was selected using Structure Harvester (Earl, 2012) by comparing the average likelihood probability estimators, calculated as ln [Pr (X|K)] for each k value, and by calculating DK based on the change in the likelihood logarithm of the data between successive values, as stated in Evanno et al. [35]. Software CLUMPP 1.1.2. [36] was used to align data between interdependent replicates for each k using the ‘‘greedy’’ algorithm with 100,000. Cultivars within a subpopulation with membership coefficients of <0.8 were considered admixed.

The hierarchical F statistic was used to estimate the proportion of genetic variance accounted for by origin and maturity group using ancestry estimates for K = 2 and calculated using the *hierfstat* R package [37].

Analysis of molecular variance (AMOVA) was performed to determine within and between-group variation using poppr.amova function in the *poppr* R package [33]. Genetic differentiation between populations was determined using the value of the phi statistic (PhiPT). The probability value used to test the significance of the variance was estimated using 1000 standard permutations.

The hierarchical F statistic and AMOVA only groups, which contains 10 or more cultivars, were included in the analysis.

## Figures and Tables

**Table 1:** Genetic diversity patterns across the groups by origin and maturity.

	No. of Cultivars	H_e_	PIC	MAF
Overall	207	0.34	0.27	0.25
Origin				
Austria	16	0.31	0.25	0.23
Croatia	38	0.28	0.23	0.21
France	22	0.29	0.23	0.21
Italy	39	0.30	0.24	0.22
Romania	16	0.29	0.24	0.21
Serbia	30	0.31	0.25	0.22
Switzerland	10	0.24	0.19	0.18
America	22	0.33	0.26	0.24
Maturity group				
MG000/00	54	0.31	0.25	0.23
MG0	60	0.33	0.26	0.24
MGI	70	0.32	0.26	0.24
MGII	23	0.32	0.25	0.23

**Table 2:** Analysis of molecular variance (AMOVA) among countries of origin and maturity groups.

Source of Variation	Country of Origin			Maturity Group		
	df	Sigma	%	df	Sigma	%
Between Populations	7	172	9.8	3	80.1	4.5
Within Populations	185	1592	90.2	203	1684.7	95.5
Total	192	1764	100	206	1764.8	100

**Table 3:** F_st_ among countries of origin and maturity groups.

Country of Origin	Austria	Croatia	France	Italy	Romania	Serbia	Switzerland	Average	Range
America	0.05	0.11	0.07	0.06	0.06	0.06	0.19	0.08	(0.05–0.19)
Austria		0.12	0.10	0.09	0.08	0.08	0.20	0.10	(0.05–0.20)
Croatia			0.15	0.15	0.15	0.12	0.23	0.15	(0.11–0.23)
France				0.12	0.13	0.10	0.24	0.13	(0.07–0.24)
Italy					0.12	0.09	0.23	0.12	(0.06–0.23)
Romania						0.09	0.25	0.12	(0.06–0.25)
Serbia							0.18	0.10	(0.06–0.18)
Switzerland								0.22	(0.18–0.25)
F_st_AVG								0.128	
**Maturity** **group**	**MG0**	**MGI**	**MGII**					**Average**	**Range**
MG000/00	0.051	0.065	0.070					0.062	(0.051–0.070)
MG0		0.030	0.045					0.042	(0.030–0.051)
MGI			0.058					0.051	(0.030–0.065)
MGII								0.058	(0.045–0.070)
F_st_AVG								0.053	

**Table 4:** Plant material by country of origin and maturity group.

Country of Origin	No.	Maturity Group	No.
Austria	16	I	70
Croatia	38	II	23
France	22	0	60
Germany	5	000/00	54
Hungary	1		
Italy	39		
America	22		
Noah Ark	8		
Romania	16		
Serbia	30		
Switzerland	10		

## Data Availability

The original contributions generated for this study are included in the article/Supplementary Material. The genotypic data analyzed in this study are available from the corresponding author upon reasonable request.

## Supplementary Materials

The following supporting information can be downloaded at: https://www.mdpi.com/article/10.3390/plants12091837/s1, Figure S1: Cluster analysis of 207 soybean cultivars using Ward’s minimum variance method based on Nei’s genetic distances. Colors represent maturity groups of cultivars.; Figure S2: Structure of analyzed soybean germplasm and its maturity groups’ representation according to geographic origin of breeding programs. Table S1: STRUCTURE results; Table S2: Number of cultivars in each cluster group according to the country of origin; Table S3. Number of cultivars in each cluster group according to their maturity group; Table S4 Plant material.
