# Supplementary figures and images for: Genetic Diversity and Population Structure of European Soybean Germplasm Revealed by Single Nucleotide Polymorphism
Source: Plants (Basel). 2023 Apr 29;12(9):1837. doi: 10.3390/plants12091837 (PMC10180984; doi:10.3390/plants12091837)

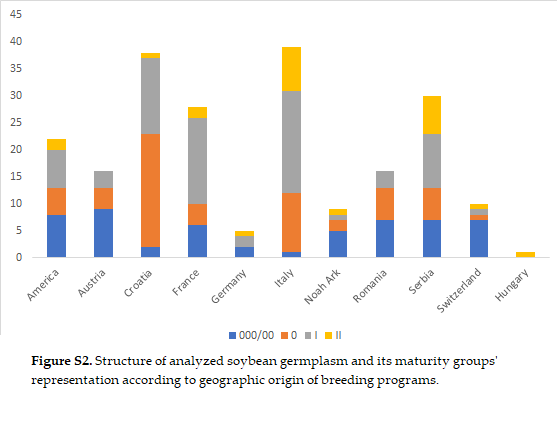

Supplement: Supplementary file 1 [file plants-12-01837-s001.zip › Supplementary_figure_S2_Structure_by_MG_revised.png]

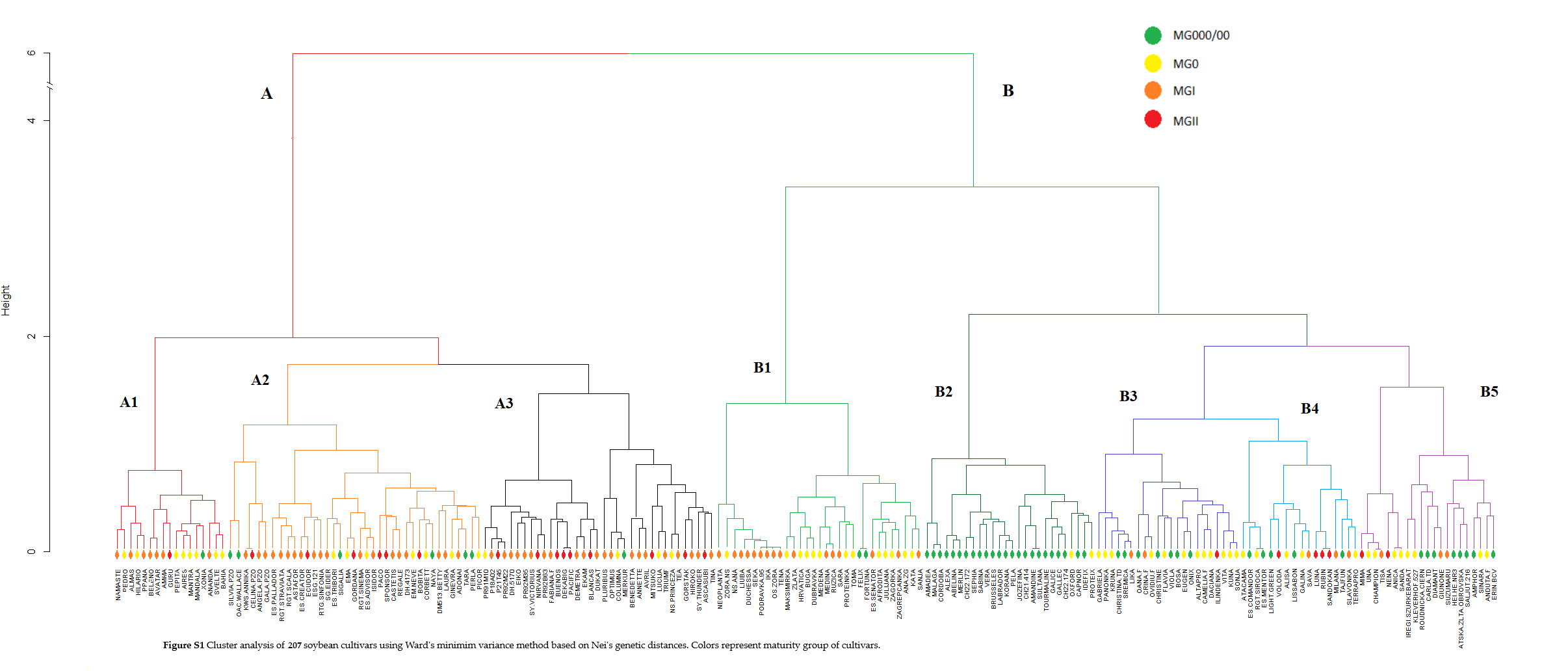

Supplement: Supplementary file 1 [file plants-12-01837-s001.zip › Supplement_figure_S1_dendrogram_by_MG_revised.png]
